# Case Report: Novel *CNGA3* compound heterozygous variants cause achromatopsia in three patients from a family

**DOI:** 10.3389/fgene.2024.1457569

**Published:** 2024-11-29

**Authors:** Xiaoqiang Zhou, Yasi Zhou, Shuijuan Wu, Xiaoling Guo, Liangfeng Yao, Xingkun Yang

**Affiliations:** ^1^ Prenatal Diagnosis Center, Foshan Women and Children Hospital, Foshan, Guangdong, China; ^2^ Foshan pulisheng Biotechnology, Foshan, Guangdong, China; ^3^ Department of Radiology, Foshan Women and Children Hospital, Foshan, Guangdong, China; ^4^ Women and Children Medical Research Center , Foshan Women and Children Hospital, Foshan, Guangdong, China

**Keywords:** achromatopsia, CNGA3 gene, genetic counseling, genetic testing, novel variant

## Abstract

This study report a novel missense variant in the *cyclic nucleotide-gated channel 3 (CNGA3)* gene identified by targeted gene panel sequencing approach in a Chinese family with achromatopsia. The proband, a 24-year-old female, with normal intelligence, motor development and speech abilities exhibited nystagmus, amblyopia, photophobia, and indistinguishable colors. In addition, the two sisters of the proband had the same clinical symptoms, which means that three patients from a family with a monochromasia clinical diagnosis. Based on the family situation, the proband came to our hospital for facilitate genetic counseling. Genetic analysis using targeted gene panel sequencing was conducted to confirm causative variants. Compound heterozygous variants, including the novel missense c.524T>A (p.Ile175Asn) and the know missense variant c.829C>T (p.Arg277Cys), were identified in *CNGA3*. These variants represent the genetic defects associated with achromatopsia in this family.

## 1 Introduction

Achromatopsia (ACHM), also known as rod monochromatism, is a congenital autosomal recessive retinal disorder that affects approximately one in 30,000 live births worldwide (Arshad et al., 2019). This condition is characterized by significant ocular abnormalities, including horizontal pendular nystagmus, photophobia, amaurosis, color blindness, severe myopia, and potential cataract development over time (Amaral et al., 2023). Symptoms typically manifest in patients during infancy or early childhood (Hirji et al., 2018). Currently, six genes, including *CNGA3* (OMIM 600053), *CNGB3* (OMIM 605080), *GNAT2* (OMIM 139340), *ATF6* (OMIM 605537), *PDE6C* (OMIM 600827), and *PDE6H* (OMIM 601190), are recognized as responsible for more than 90% of ACHM cases (Abdelkader et al., 2018; Liang et al., 2015). Molecular genetic analysis of ACHM patients reveals that, despite the discrepancy in the prevalence of *CNGA3* gene variants among different ethnic groups, the majority of ACHM patients carry pathogenic variants in the cyclic nucleotide-gated channel 3 (*CNGA3*) gene (Solaki et al., 2022).

Variants in the *CNGA3* gene, located on chromosome 2q11, which encodes the alpha subunit of the cone photoreceptor cGMP-gated cation channel, are the cause of achromatopsia 2 (ACHM2; OMIM 216900) (Dubis et al., 2014; Fischer et al., 2020). Activation of the cyclic nucleotide-gated (CNG) channel, a member of the superfamily of voltage-gated ion channels in photoreceptors, is crucial for color vision and visual acuity (Kaupp and Seifert, 2002; Brown et al., 2006; Gerhardt et al., 2023a). The channel comprises two structurally related subunit types, the A and B subunits (Napolitano et al., 2021). The rod CNG channel is composed of *CNGA1* and *CNGB1* while cone CNG channel is composed of *CNGA3* and *CNGB3*, indicating that the *CNGA3* gene encodes one of a family of alpha subunits essential for forming CNG ion channels involved in visual signal transduction (Gerhardt et al., 2023b).

The symptoms of ACHM include day blindness, nystagmus, photophobia, indistinguishable colors, normal funduscopy and rod monochromacy (Nishiguchi et al., 2005; Zheng et al., 2022). Genetic testing is necessary because variants in the other five genes can cause similar symptoms that cannot be distinguished clearly by phenotype alone.

In this study, a novel missense variant (c.524T>A p.Ile175Asn) of *CNGA3* identified in a Chinese family with achromatopsia in conjunction with clinical data was first reported.

## 2 Case presentation

### 2.1 Subjects

The three patients in a Chinese family with *CNGA3* variants was investigated for this genetic study. Medical histories of the family were collected, and an initial diagnosis was made.

### 2.2 Clinical phenotype

**FIGURE 1:**
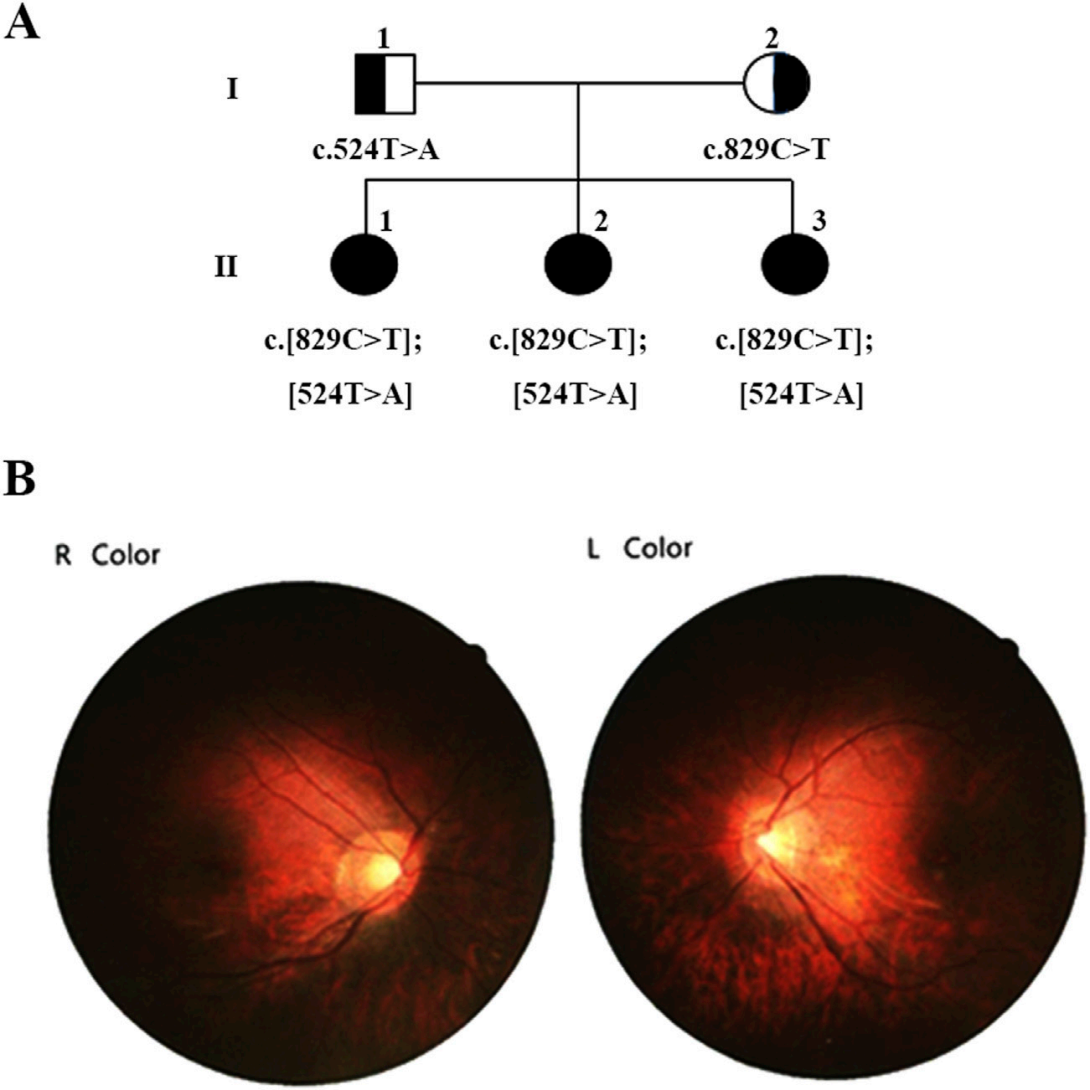
Family pedigree demonstrating the segregation of causative variants. **(A)** Pedigree of the family showing segregation of the variants in the gene *ANGA3*. The different mutated alleles were inherited from each parent, resulting in compound heterozygous *ANGA3* variants. Available genotypes are indicated below the symbols. **(B)** Fundus Oculi photography of proband II:1.

The proband (Figure 1A, II-1), a 24-year-old female, exhibited nystagmus at the age of 1, amblyopia at 2 years old, photophobia and indistinguishable colors. However, the proband exhibited normal fundus oculi without hypopigmentation (Figure 1B) and normal development in terms of intelligence, motor skills, and language. The clinical diagnosis was ACHM.

The proband sought genetic counseling to ensure the birth of a child with a normal phenotype. After obtaining informed consent, we conducted a detailed investigation of the proband’s family situation.

Her two younger sisters (Figure 1A, II-2 and II-3), 12 and 11 years old, exhibit nystagmus, amblyopia, photophobia, and color discrimination issues, with the onset of symptoms occurring at an age similar to the proband, and were also diagnosed with ACHM. The proband has myopia of −8.00 diopters, while her sister has myopia of −7.00 diopters. The best-corrected visual acuity of both the proband and her sister is 0.8. However, their parents have normal vision and no abnormal ophthalmic symptoms.

The blood samples were collected from five members of the family, and DNA was extracted following PCR amplification. The targeted gene panel sequencing was performed exclusively on the proband. For the other family members, Sanger sequencing was utilized to confirm the variants identified in the proband.

## 3 Methods

### 3.1 Targeted gene panel sequencing approach

A targeted gene panel sequencing approach was employed, capturing and sequencing 983 genes associated with ophthalmic diseases using the Illumina Nextseq 500 platform. The average coverage depth of sequence was >200X and coverage greater than 20X accounted for 99.5%. The sequencing data were analyzed by an in-house bioinformatic methods (AmCare Genomics Lab, Guangzhou, China) (Qi et al., 2020).

### 3.2 Database retrieval and analysis

We retrieved internal database, for instance, dbSNP (http://www.ncbi.nlm.nih.gov/snp), 1000 Genomes Project (http://browser.1000genomes.org), Exome Aggregation Consortium (ExAC) (http://exac.broadinstitute.org/) and such population database, labeling single nucleotide polymorphism (SNP) and low frequency benign variation. Subsequently, we searched Human Gene Mutation Database (HGMD) (http://www.hgmd.org), OMIM (http://www.omim.org), ClinVar (http://www.ncbi.nlm.nih.gov/clinvar) and variants related literature for analysis. The protein structure was predicted byalphafold3 (https://alphafoldserver.com/). The three-dimensional (3D) protein structures of wild-type and mutant *CNGA3* proteins were predicted using Pymol.

### 3.3 Variants classification

We predicted the conservation, pathogenicity or harmfulness of variants using online predictive software such as PolyPhen-2 (http://genetics.bwh.harvard.edu/pph2), SIFT (http://sift.jcvi.org) and Mutation Taster (http://www.mutationtaster.org). Finally, variants were classified according to the guideline of the American College of Medical Genetics and Genomics (ACMG) and verified by Sanger sequencing.

## 4 Results

**FIGURE 2:**
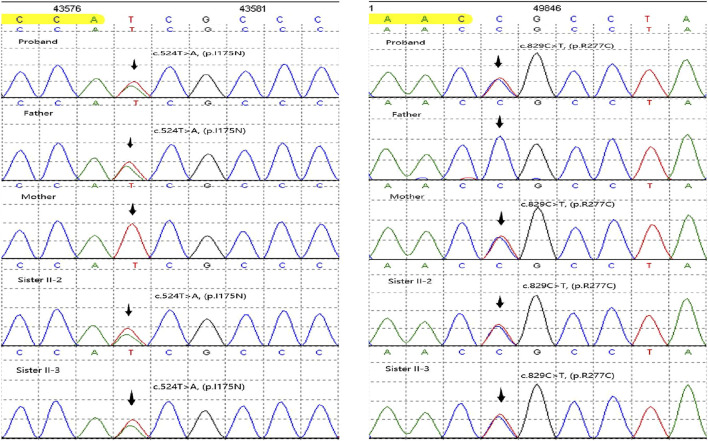
Results of Sanger sequencing of *CNGA3* variants in this family.

We initially identified compound heterozygous variants, NM_001298: c.829C>T (p.Arg277Cys) and NM_001298: c.524T>A (p.Ile175Asn) in *CNGA3* gene in the proband (Figure 1A, II:1) by targeted gene panel sequencing (Table 1). Then segregated the disease status, these two variants were later confirmed in the proband’s parents (Figure 1A, I:1 and I:2) and the proband’s younger sisters (Figure 1A, II:2, II:3) respectively by Sanger sequencing (Figure 2). The heterozygous *CNGA3* gene variant c.829C>T (p.Arg277Cys) was found in the proband’s asymptomatic mother (Figure 1A, I:2) and the affected proband (Figure 1A, II:1) and her two sisters (Figure 1A, II:2, and II:3). The variant c.524T>A (p.Ile175Asn) in *CNGA3* gene was found in the proband’s asymptomatic father (Figure 1A, I:1) and the affected proband (Figure 1A, II:1) and her two sisters (Figure 1A, II:2, and II:3). This compound heterozygous variants co-segregated with the phenotype of achromatopsia in this family (Figure 2).

**TABLE 1:** Classification status of the causative variants in the gene *CNGA3*.

Exon	Variants	SIFT	Variant taster	Polyphen-2	Conservation	Allele frequency	ACMG	Parental origin
6	c.524T>A p.Ile175Asn	Damaging 0.000	Disease causing 0.999	Probably damaging 0.998	Yes	—	Uncertain significance	Paternal
8	c.829C>T p.Arg277Cys	Damaging 0.000	Disease causing 0.999	Probably damaging 1.000	Yes	<0.001	Pathogenic	Maternal

**FIGURE 3:**
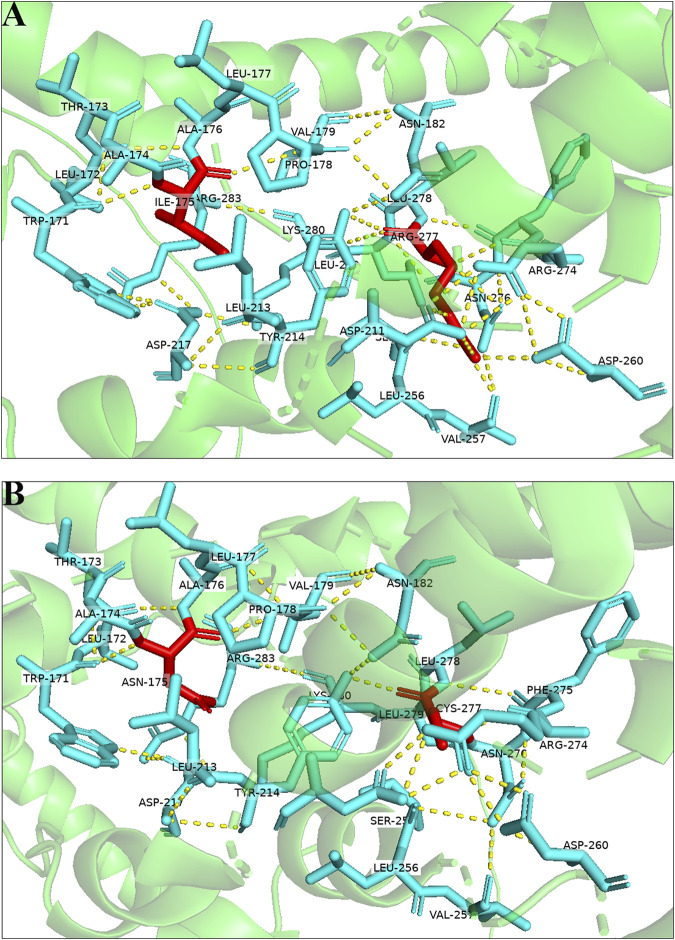
Three-dimensional structures of the wild-type and mutant proteins: **(A)** Wild-type protein; **(B)** mutant proteins of the variant.

3D protein modeling of the wild-type and mutated *CNGA3* protein, based on NP_001289.1, revealed significant structural changes. The p.Ile175Asn variant resulted in the formation of a new hydrogen bond between Ala176 and Val179, while the p.Arg277Cys variant led to the disruption of hydrogen bonds with Asp211 and Val257, occurring in a tightly folded β-sheet region, suggesting potential functional impacts on the *CNGA3* protein (Figure 3).

**FIGURE 4:**
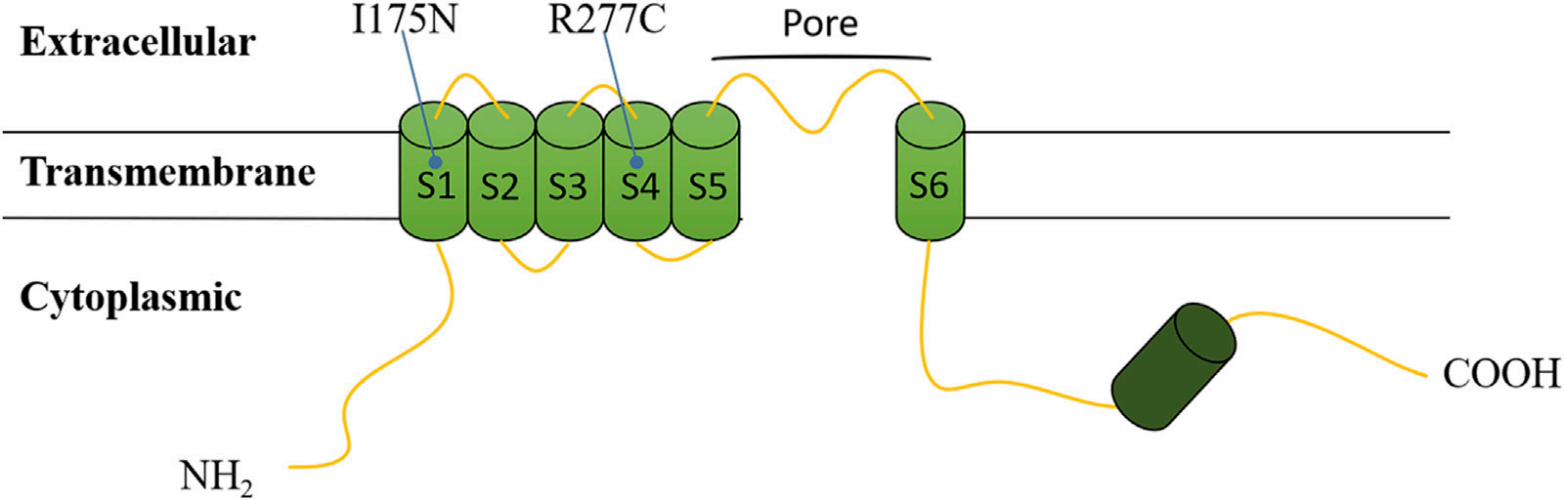
Topological model of the *CNGA3* polypeptide based on Uniprot. Variants are depicted at their respective positions within the polypeptide.

The *CNGA3* polypeptide is topologically composed of six transmembrane helices (S1-S6), with the variants that we found located in the pore region between S5 and S6, a cyclic nucleotide-binding domain (CNBD), and a C-linker between S6 and CNBD (Figure 4) (Chen et al., 2015; Reuter et al., 2008).

## 5 Discussion

Variants of the *CNGA3* gene are part of the causes of ACHM. Previous studies and variants documented in HGMD indicated that most of them are missense variants (Reuter et al., 2023), suggesting that the CNG channel cannot tolerate substitutions. In addition, differential diagnosis may be made between ACHM and other *CNGA3*-associated retinopathy, such as cone dystrophy or cone-rod dystrophy (CORD), Leber congenital amaurosis (LCA), and oligocone trichromacy (OCT) (Sun et al., 2020). The key to identify these diseases lies in the clinical phenotype and auxiliary examination. The course of CORD typically progresses over time, whereas ACHM remains stable. LCA may involve fundus oculi changes in infants and the symptom of OCT does not include color blindness (Sun and Zhang, 2019).

In this study, we detected two missense variants, including a novel variant, c.524T>A (p.Ile175Asn), which was first reported in association with complete ACHM. The c.524T>A variant in exon 6 of *CNGA3* gene is a missense variant, identified as an uncertain significant variant by the ACMG guideline (Richards et al., 2015). It has never been reported in controls and locates in a critical functional domain of *CNGA3*, the S1 transmembrane helix. It means we can use moderate evidence PM1 in ACMG guideline. Moreover, it is predicted to be deleterious by different variant prediction tools online. It means we can use supporting evidence PP3 in ACMG guideline. In conclusion, the evidence of pathogenicity of the c.524T>A variant is PM1 and PP3 in ACMG, so, it is assessed as uncertain significant variant.

In *CNGA3*, c.829C>T is located in S4 transmembrane helix, and identified as pathogenic variant by ACMG. We compared the phenotypes of our patients with those cases carrying the c.829C>T (p.Arg277Cys) variant reported in previous studies (Table 2). Most patients diagnosed with ACHM in these studies exhibited photophobia and nystagmus, consistent with our findings. This comparison highlights the recurrent association of these symptoms with ACHM, reinforcing their diagnostic value. Therefore, for pediatric patients presenting with nystagmus and/or photophobia, accompanied by vision impairment that is not correctable, it is crucial to consider genetic retinal diseases in the differential diagnosis. This is especially important for cases with unexplained vision loss. Autosomal recessive genetic disorders, including ACHM, exhibit a complex relationship between phenotype and genotype. This complexity arises primarily from factors such as which of the two variant alleles retains essential functional activity in compound heterozygotes, whether the two variant alleles demonstrate synergistic effects, and whether they can complement each other. Some studies classify ACHM into complete achromatopsia (cACHM) and incomplete achromatopsia (iACHM) (Table 2). The differentiation between iACHM and cACHM is achieved through extended electrophysiological techniques, specifically full-field and multifocal electroretinography (ERG) (Zobor et al., 2017). Regrettably, the clinical data in our case is inadequate to definitively categorize it as either cACHM or iACHM. Nonetheless, previous studies have showed that most patients harboring the c.829C>T (p.Arg277Cys) heterozygous variant exhibit cACHM, with two cases of homozygous variants also presenting as cACHM (Wissinger et al., 2001; Zobor et al., 2017). However, the small sample size limits the observation of a correlation between the c.829C>T (p.Arg277Cys) variant and the absence of detectable photopic responses in cACHM patients.

**TABLE 2:** Clinical presentation of patients with *CNGA3* variants.

Patient origin	^a^Clinical diagnosis	*CNGA3* genotypes	Photophobia	Nystagmus	Parents	Siblings
This case (proband II:1)	ACHM	c.829C>T (p.Arg277Cys) c.524T>A (p.Ile175Asn) (n = 1)	Yes	Yes	normal	ACHM (2/2)
Lam et al. (2011)	ACHM	c.829C>T (p.Arg277Cys) c.1580T>G (p.Leu527Arg) (n = 1)	Yes	Yes	normal	ACHM (1/2)
Wissinger et al. (2001)	cACHM (4/9)	The missense variant c.829C>T (p.Arg277Cys) is observed in several heterozygous genotypes (n = 9)	Yes	Yes	None	None
	iACHM (2/9)		Yes	Yes	None	None
Zobor et al. (2017)	cACHM	c.829C>T (p.Arg277Cys) Homozygous genotypes (n = 2)	None	None	None	Two patients were siblings
	cACHM	c.661C>T (p.Arg221*) c.829C>T (p.Arg277Cys) (n = 1)	None	None	None	None
	iACHM	c.829C>T (p.Arg277Cys) c.1687C>T (p.Arg563Cys) (n = 1)	None	None	None	None

^a^ The difference between cACHM, and iACHM, patients is that photopic responses were nondetectable in cACHM, patients, while residual cone responses could be observed in iACHM, patients (Zobor et al., 2017). cACHM = complete achromatopsia; iACHM = incomplete achromatops.

It has previously been reported in patients with achromatopsia (Reuter et al., 2008; Li et al., 2014) and established *in vitro* functional studies, supporting a significant reduction in the availability of cone photoreceptor cyclic nucleotide-gated channels on cell surface (Liu and Varnum, 2005). It means we can use strong evidence PS1 and PS3 in ACMG guideline. In addition, it is conserved across multiple species and located in a critical functional domain of *CNGA3*. It means we can use moderate evidence PM1 in ACMG guideline. Besides, it shows an extremely low frequency in controls and is predicted to be deleterious by multiple prediction software online. It means we can use moderate evidence PM2 and supporting evidence PP3 in ACMG guideline. In conclusion, the evidence of pathogenicity of the c.829C>T variant is PS1, PS3, PM1, PM2 and PP3 in ACMG. So, it is assessed as pathogenic variant.

Genetic counseling is necessary for the three affected sisters. If any of the husbands of the three sisters carry one of the pathogenic variants in the *CNGA3* gene, it is recommended to utilize *in vitro* fertilization (IVF) in conjunction with preimplantation genetic testing for monogenic disorders (PGT-M) to prevent the transmission of this genetic disease to their offspring because the incidence rate is 25 percent.

The complete concordance between genotype and phenotype in this family provides sufficient evidence that the two compound heterozygous variants, c.829C>T and c.524T>A, are the disease caused variants for achromatopsia in this family. However, the only drawback lies in the absence of functional experimental verification.

## 6 Conclusion

In this report, compound heterozygous *CNGA3* variants, c.829C>T (p.Arg277Cys) and c.524T>A (p.Ile175Asn), which co-segregated with the phenotype in this family. To our knowledge, the c.524T>A (p.Ile175Asn) variant was first reported in this study. Therefore, our case extends the variant spectrum of ACHM.

## Data Availability

The original contributions presented in the study are included in the article/supplementary material, further inquiries can be directed to the corresponding authors.
